# Effects of Three Different Modes of Resistance Training on Appetite Hormones in Males With Obesity

**DOI:** 10.3389/fphys.2022.827335

**Published:** 2022-02-21

**Authors:** Ali Ataeinosrat, Marjan Mosalman Haghighi, Hossein Abednatanzi, Mohammad Soltani, Abbass Ghanbari-Niaki, Akbar Nouri-Habashi, Sadegh Amani-Shalamzari, Ali Mossayebi, Mitra Khademosharie, Kelly E. Johnson, Trisha A. VanDusseldorp, Ayoub Saeidi, Hassane Zouhal

**Affiliations:** ^1^Department of Physical Education and Sport Science, Science and Research Branch, Islamic Azad University, Tehran, Iran; ^2^Faculty of Medicine and Health, The University of Sydney, Sydney, NSW, Australia; ^3^Department of Biological Sciences in Sport, Faculty of Sports Sciences and Health, Shahid Beheshti University, Tehran, Iran; ^4^Exercise Biochemistry Division, Faculty of Sport Sciences, University of Mazandaran, Babolsar, Mazandaran, Iran; ^5^Department of Exercise Physiology and Corrective Movements, Faculty of Sport Sciences, Urmia University, Urmia, Iran; ^6^Department of Exercise Physiology, Faculty of Physical Education and Sports Science, Kharazmi University, Tehran, Iran; ^7^Department of Kinesiology, College of Health Sciences, University of Texas at El Paso, El Paso, TX, United States; ^8^Department of Physical Education, Faculty of Literature, Kosar University of Bojnord, Bojnord, Iran; ^9^Department of Kinesiology, Coastal Carolina University, Conway, SC, United States; ^10^Department of Exercise Science and Sport Management, Kennesaw State University, Kennesaw, GA, United States; ^11^Department of Physical Education and Sport Sciences, Faculty of Humanities and Social Sciences, University of Kurdistan, Sanandaj, Iran; ^12^Laboratoire Mouvement, Sport, Santé (M2S), University of Rennes, Rennes, France; ^13^Institut International des Sciences du Sport (2I2S), Iroduer, France

**Keywords:** obesity, appetite hormones, strength, interval training, resistance training

## Abstract

**Purpose:**

This study explored the effect of three different modes of resistance training on appetite hormones [leptin, ghrelin, cholecystokinin (CCK), glucagon-like peptide-1 (GLP-1), and peptide tyrosine–tyrosine (PYY)], cardiometabolic and anthropometric measures in males with obesity.

**Methods:**

Forty-four males with obesity (age: 27.5 ± 9.4 yrs.; mean weight: 93.2 ± 2.2 kg, body mass index: 32.9 ± 1.2 kg/m^2^) were randomized to traditional resistance training (TRT, *n* = 11), circuit resistance training (CRT, *n* = 11), interval resistance training (IRT, *n* = 11) or control (C, *n* = 11) groups. All resistance training groups received 50 min of supervised training per session, three days per week, for 12 weeks. Measurements were taken at baseline and after 12 weeks of training.

**Results:**

Plasma levels of leptin, ghrelin, CCK, and PYY decreased significantly in all three different modalities of resistance training groups when compared to the control group (*p* < 0.05). GLP-1 increased significantly in both CRT and IRT groups compared to TRT and C groups (*p* < 0.05). Glucose-dependent insulinotropic polypeptide decreased significantly in CRT and IRT groups compared to the C group (*p* < 0.05). Adiponectin levels increased significantly in all resistance training groups compared to the C group (*p* < 0.05).

**Conclusion:**

Overall, CRT and IRT protocols had the greatest impact on appetite hormones compared to individuals who engaged in TRT or did not exercise (C).

## Introduction

Obesity is a global crisis that affects all ages and socioeconomic groups, and it is one of the major health concerns of the 21st century. According to the World Health Organization estimates, more than 1.9 billion adults aged 18 years and older are overweight, and of those, over 650 million are obese (Haththotuwa et al., 2020). Furthermore, it has been shown that obesity appears with increasing prevalence among younger populations, leading to an increased risk of chronic diseases, such as metabolic, cardiovascular diseases, and some forms of cancer (including breast and endometrial cancer), leading to early mortality (Van Gaal et al., 2006).

An unhealthy lifestyle, including low levels of physical activity and high quantities of food intake, is one of the main key factors accelerating the expansion of obesity (Kim et al., 2019). The hypothalamus integrates the neuronal and hormonal signals and controls eating behaviors, satiety, and caloric intake (Zouhal et al., 2019). Several hormones affecting the brain centers are synthesized and released from peripheral tissues, including intestinal and adipose tissues (adipocytes; Berthoud and Morrison, 2008). The main hormones regulating appetite and satiety are leptin, ghrelin, glucagon-like peptide-1 (GLP-1), peptide tyrosine–tyrosine (PYY), glucose-dependent insulinotropic polypeptide (GIP), and cholecystokinin (CCK; Zanchi et al., 2017).

A systematic review including a 1-year follow-up showed that exercise alone had minimal weight loss effects (Franz et al., 2007), while other research has reported that individuals who remain in a caloric deficit consistently during a 6-month time period may experience 10% weight loss (Redman et al., 2007). Additionally, in a comprehensive investigation by Ross and colleagues demonstrated that exercise-induced weight loss reduced total fate and improved cardiovascular fitness more than equivalent diet-induced weight loss (Ross et al., 2000). These studies concur that longer bouts of exercise (commonly, a daily 700-calorie energy expenditure) have been associated with a greater contributions to weight loss. Regular exercise has been shown to facilitate weight management by increasing energy expenditure which influences appetite hormones and overall daily energy expenditure irrespective of adiposity and sex (Zouhal et al., 2019, 2020). On the other hand, previous studies have shown that physical inactivity disrupts the mechanisms responsible for regulating appetite (Hopkins and Blundell, 2016). In contrast, physical activity in sedentary individuals with obesity has been shown to regulate appetite (Rocha et al., 2016). The effectiveness of resistance training for weight control in normal-weight individuals in the absence of calorie restriction is controversial. Although molecular mechanisms of appetite regulation following exercise are still unknown, and few studies are available on the effect of resistance training on appetite regulation and the hormones involved in appetite regulation (Neary et al., 2004; Guelfi et al., 2013).

In a weight loss program, resistance training is recommended to maintain or improve muscle mass to increase basal metabolism as well as reduce fat (Balaguera-Cortes et al., 2011; Guelfi et al., 2013; Douglas et al., 2017). The resistance training was conducted as traditional, circuit, and interval models. It has been shown that traditional resistance training interventions reduce energy intake (Balaguera-Cortes et al., 2011; Guelfi et al., 2013; Douglas et al., 2017). Research has shown 12 weeks of circuit resistance training [3 sessions per week at 60% of 1 repetition max (1-RM)] in overweight adolescents resulted in food intake decrements along with weight loss (Tavassoli et al., 2014). However, the effects of interval resistance training (IRT), a new exercise model, on weight loss are less well known (Moro et al., 2020). Given obese individuals have less time to exercise and cannot lift heavy weights, IRT leads to more physiological loads to be exerted on the subjects in less time duration (Moro et al., 2020). Most resistance training interventions have demonstrated decrements of regulatory appetite markers (Kraemer et al., 2004; Ghanbari-Niaki, 2006; Ballard et al., 2009), with a few showing opposite results (Martins et al., 2007; Kelishadi et al., 2008; Hagobian et al., 2009; Konopko-Zubrzycka et al., 2009; Ueda et al., 2013). However, to our knowledge, no studies have compared the effects of different modes of resistance training on appetite-regulating hormones. Given the importance of appetite and energy intake on energy balance, understanding how these hormones change with different resistance training methods has important implications for individuals with obesity. Therefore, this study aimed to compare traditional, interval, and circuit resistance training on appetite-regulating hormones in males with obesity.

## Materials and Methods

### Study Subjects

Forty-four males with obesity [mean age: 27.50 ± 9.4 yrs. and body mass index (BMI): 32.9 ± 1.2 kg/m^2^] were recruited through advertisements (poster, email, and social media platforms). Inclusion criteria were: age between 23 and 32 yrs., BMI > 30 kg m^−2^, waist-to-height ratio > 0.6, being sedentary, which was defined as less than 1 h of physical activity per week in the past 12 months, not diagnosed with chronic illnesses (e.g., cardiovascular disease, diabetes, and hypertension), non-smokers, not undertaking hormonal or mental therapy, and were not drinking alcohol. Subjects who took dietary supplements and medications that affect the metabolism of muscle or adipose tissue (e.g., amino acids, beta-blockers, beta-agonists, calcium channel blockers, and corticosteroids) were excluded from the study. Inclusion criteria were assessed by a physician and also using the Physical Activity Readiness Questionnaire and medical health/history questionnaires (Thomas et al., 1992). All participants completed an informed consent form. The study was approved by the Research and Ethics Committee of the Islamic Azad University (Ethics code: IR-IAU1397-16) and performed according to the latest revision of the Declaration of Helsinki (Nathanson, 2013).

### Study Design

All participants were thoroughly acquainted with all testing and procedures before obtaining baseline measurements. Subsequently, participants based on weight and BMI were randomized into one of the four groups: circuit resistance training (CRT; *n* = 11), traditional resistance training (TRT; *n* = 11), interval resistance training (IRT; *n* = 11), or control (C; *n* = 11). Study measurements were collected at two time points; baseline (48 h before starting preparatory phase) and after 12 weeks (48 h after the last training session) at the same time of the day (within ~1 h) and under the same environmental conditions (~20°C heat and ~ 55% humidity). In addition, the participants were asked to continue their usual dietary habits for the study duration.

### Preparatory Phase

All subjects performed 1 week of resistance training under supervision, consisting of three exercise sessions, for familiarization before the primary training intervention. This phase allowed instruction relating to correct lifting techniques, familiarizing all exercises and equipment, and ensuring that the participants initiated the study at a comparable starting level in exercise familiarization and is commonly used in training interventions of this type.

### Resistance Training Interventions

All resistance training sessions consisted of 70 min of supervised exercise that included a 10-min warm-up, 10-min cool-down, and 50-min exercise session. All three resistance training groups utilized the same exercises, which were back squat, lat pulldown, leg press, chest press, leg extension, leg curls, lateral raise, standing calf raise, biceps curl, and triceps pushdown (Table 1). The CRT protocol included three circuits of 10 exercises for each circuit, at an intensity of 50% of 1-RM with 14 repetitions of each exercise. Participants in this group performed these exercises with a minimum rest (<15 s) interval between exercises with a rest period of 3 min between sets (Saeidi et al., 2020). The TRT group performed the same exercises at 50% of 1RM and 14 repetitions for three sets and 30 s rest interval between exercises and 1.5 min rest between sets (Saeidi et al., 2020). The IRT group received the same exercises with 50% of 1 RM, and 14 repetitions were followed with active rest of 25% of 1RM and 14 repetitions; subjects performed each exercise in two sets (Saeidi et al., 2020). The periodized resistance modes were adapted from previous studies (Baechle and Earle, 2008). During this study, the control group (C) did not participate in any exercise training. Exercises were performed on variable resistance machines (Hoist equipment, San Diego, United States; Table 1), and the resistance training mode volumes equated using the following formula (Saeidi et al., 2020):


Resistance training volume=number of sets×repetitions×lifted weight.

**Table 1:** Exercises, sets, repetitions, and rest intervals between exercises and sets and a load of exercise for each weekly session in the TRT, CRT, and IRT groups.

Duration	12 Weeks	12 Weeks	12 Weeks
**Program type**	**TRT**	**CRT**	**IRT**
Session Overview	Exercises: back squat, lat pulldown, leg press, chest press, leg extension, leg curls, lateral raise, standing calf raises, biceps curl, and triceps pushdown Sets: 3 Repetitions: 14 Rest interval between exercises: 30 s Rest between each set: 1.5 min Load: 50% of 1RM	Exercises: back squat, lat pulldown, leg press, chest press, leg extension, leg curls, lateral raise, standing calf raises, biceps curl, and triceps pushdown Sets: 3 Repetitions: 14 Rest interval between exercises: 15 > seconds Rest between each set: 3 mines Load: 50% of 1RM	Exercises: back squat, lat pulldown, leg press, chest press, leg extension, leg curls, lateral raise, standing calf raises, biceps curl, and triceps pushdown Sets: 2 Repetitions: 14 Rest interval between exercises: active rest with 25% of 1RM and 14 repetitions Load: 50% of 1RM

TRT, Traditional resistance training; CRT, Circuit resistance training; IRT, Interval resistance training; 1RM, One maximum repetition.

The volume and loading of the three resistance interventions were similar between the groups (50%). Although participants in the IRT group performed the exercises at 50% of 1RM and 25% at rest, we reduced the number of sets to two to equalize loading between the three groups:


$$\mathrm{IRT}\;\left(\mathrm{Exercise load}:50\%+\mathrm{rest exercise load}\;25\%\right)\times 2\;\mathrm{sets}=150\mathrm{AU}.$$

$$\mathrm{CRT}\;\left(\mathrm{Exercise load}:50\%+\mathrm{rest exercise load}\;0\%\right)\times 3\;\mathrm{sets}=150\mathrm{AU}.$$

$$\mathrm{TRT}\;\left(\mathrm{Exercise load}:50\%+\mathrm{rest exercise load}\;0\%\right)\times 3\;\mathrm{sets}=150\mathrm{AU}.$$

### Strength Testing

Strength testing occurred 24 h after the assessment of body composition and drawing of venous blood samples, which was repeated every 4 weeks. A one-repetition maximum estimation was performed to determine training intensity for the resistance training sessions. Before testing, research personnel explained the purpose of each test, attendant risks, possible discomforts, and the participants’ responsibilities. All participants were instructed to refrain from consuming alcohol (for 48 h), caffeinated drinks (for 12 h), and food (for 2 h) before the testing sessions; however, participants’ regular water consumption was allowed. Following a light warm-up, with both general and specific components, the participants underwent strength testing for all exercises, which included (back squats, lat pulldown, leg press, chest press, leg extension, leg curls, lateral raise, standing calf raise, biceps curl and triceps pushdown) by using variable resistance machines (Hoist equipment, San Diego, United States). The participants performed two attempts, and their highest lifted weight and number of repetitions were recorded. The number of repetitions to fatigue did not exceed 10. There was a 5 min rest period between attempts. Maximal strength was estimated from these assessments using the Brzycki equation: 1RM = weight/(1.0278–0.0278 × reps) (Brzycki, 1993). All training sessions were performed under the supervision of an exercise physiologist.

### Anthropometry and Body Composition

Anthropometric and body composition measures (body weight, height, and BMI) were evaluated using standard techniques before and after the training protocol (Pearson et al., 2018). Bodyweight (nearest 0.1 kg) and height (Nearest 0.1 cm) were measured with light clothing and no footwear after an overnight fast using a digital scale and a stadiometer (Medigate Company Inc., Dan-dong Gunpo, Korea). The BMI was calculated by dividing body weight (kg) by the square of their height (m^2^). A bio-impedance analyzer (Medigate Company Inc., Dan-dong Gunpo, Korea) measured body fat.

### Blood Sampling and Analysis

Fasting blood samples (20 ml) were collected from the antecubital vein using standard procedures following a 12-h overnight fast. Following completion of blood sampling, the samples were centrifuged at 3000 rpm for 10 min, and the plasma was stored at −70 ° C until analysis. Plasma GLP-1 [Abcam, United States, Cat No: ab229413: sensitivity 12 pg/ml, intraassay coefficients of variability (CV) 7% and the inter-assay CV 9%], GIP (Mybiosource, United States, Cat No: MBS9136990: sensitivity 23.9 pg/ml, intraassay CV <10% and the inter-assay CV 15%), Leptin (Abcam, United States, Cat No: ab108879: sensitivity = 80 pg/ml, intraassay CV 3.8%and the inter-assay CV 9.9%), Adiponectin (Abcam, United States, Cat No: ab108786: sensitivity = 0.7 ng/ml, intraassay CV 3% and the inter-assay CV 8.3%), total Ghrelin (eBioscience, Vienna, Austria, Cat no: BMS2192): sensitivity 11.8 pg/ml, intraassay CV 6% and the inter-assay CV 8.5%, total PYY (Abcam, United States, Cat No: ab255727: sensitivity = 8.4 pg/ml, intraassay CV 11.7% and the inter-assay CV 12.2%), CCK (MyBioSource, United States, Cat No: MBS165254: sensitivity 0.1 ng/dl, intraassay CV <8%and the inter-assay CV <10%), concentration were measured using an ELISA.

### Nutrient Intake and Dietary Analysis

Three-day food records (2 weekdays and 1 weekend day) were obtained before and following the study to assess changes in habitual dietary intake over time (Thomas et al., 2016). In addition, each food item was individually entered into Diet Analysis Plus version 10 (Cengage, Boston, MA, United States) and total energy consumption, as well as the amount of energy derived from proteins, fats, and carbohydrates (Thomas et al., 2016) was calculated and recorded (Table 2).

**Table 2:** Pre- and post-values of nutritional intake in the four study groups.

	Control		TRT		CRT		IRT	
	Pre	Post	Pre	Post	Pre	Post	Pre	Post
Energy (kcal/d)	2,266 ± 68	2,272 ± 85	2,285 ± 112	2,323 ± 151	2,263 ± 128	2,352 ± 168	2,282 ± 178	2,381 ± 185
CHO (g/d)	280 ± 13.4	282 ± 20.3	278.4 ± 78.1	280 ± 68.5	283 ± 47.6	286 ± 18.2	288 ± 19.6	291 ± 20.1
Fat (g/d)	81.2 ± 11	80 ± 9.82	85.5 ± 10.7	87 ± 14.2	80.4 ± 15.4	87.1 ± 12.2	79.8 ± 9.87	87.2 ± 14.3
Protein (g/d)	103 ± 10	105 ± 12.3	100 ± 13.5	105 ± 10.6	102 ± 16.8	106 ± 12.7	103 ± 14.5	108 ± 13.5

*Data presented as mean (±SD), TRT, Traditional resistance training; CRT, Circuit resistance training; IRT, Interval resistance training; CHO, carbohydrate; g/d, gram (s)/day*.

### Statistical Analysis

All statistical analyses were conducted by SPSS statistical software (Version 24.0; SPSS, Inc., Chicago, IL). Normality of data evaluated with the Shapiro–Wilk test. The Leven test showed that the homogeneity of variances was present (*p* > 0.05). Baseline data were compared between the four groups using one-way ANOVA tests and Tukey’s post-hoc tests. The two Repeated measures test of ANOVA (Group * time) were used to compare the differences in the study’s variables between the four groups. If an ANOVA test determined significant differences, pairwise comparisons (planned comparisons) were used to determine which mean differences were statistically significant. Partial eta-squared (*p*^2^) was used to assess the effect size (ES). Statistical significance was established at *p* ≤ 0.05. Before analysis, the sample size was designed to detect a difference among study variables with a 95% confidence interval (CI) and 80% or greater power value. Data are presented as the mean ± standard deviation (mean ± SD).

## Results

**Figure 1:**
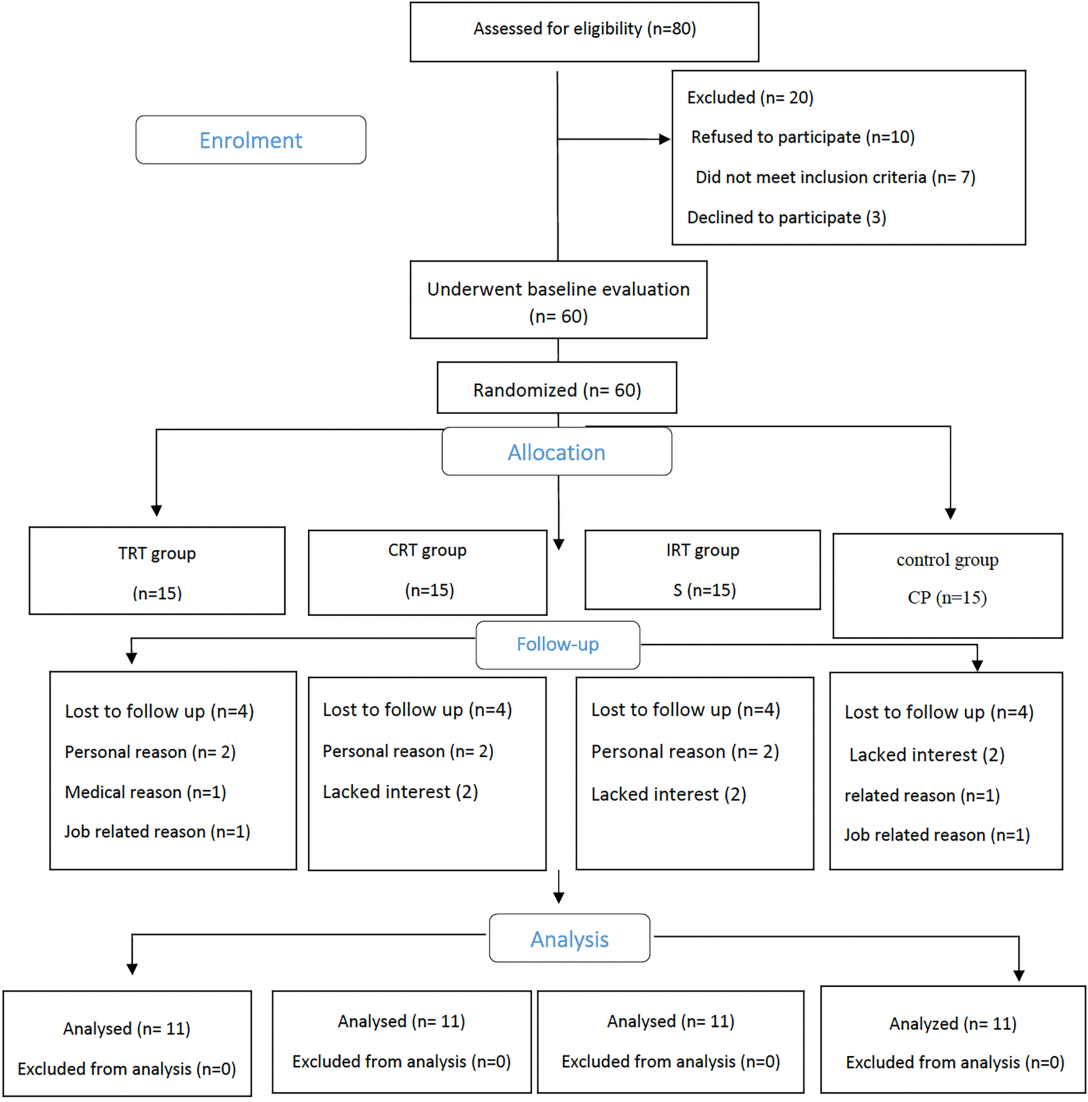
Study flow chart. Traditional resistance training (TRT), Circuit resistance training (CRT), and Interval resistance training (IRT) groups.

Participant flow through the study is shown in the CONSORT flowchart (Figure 1). Overall, of 80 participants screened in the study, 20 did not meet the inclusion criteria, 60 received the allocated intervention, and 44 attended the 12-week assessment, resulting in a drop-out rate of 26.6%. Adherence to the intervention was 74% of sessions in the training groups. There were no significant differences in baseline characteristics between the groups (*p* > 0.05).

### Anthropometry and Body Composition

There was a significant group x time interaction for changes in weight, BMI, and fat mass (*p* < 0.05) between resistance and control groups. Weight was significantly reduced in the CRT and IRT groups (*p* < 0.05), but not in the TRT group (*p* > 0.05) compared to the control group (Table 3). BMI decreased only in the IRT and not in the other training groups when compared to the control group (*p* < 0.05; Table 3). Fat mass was reduced in all three resistance groups when compared to the control group (*p* < 0.05; Table 3). The SBP decreased significantly in both CRT and IRT groups compared to the control groups (*p* < 0.05). However, this reduction was significantly greater in the IRT group than the TRT (*p* < 0.05; Table 3). There were no significant differences in DBP in all resistance training groups compared to the control group (*p* < 0.05; Table 3).

**Table 3:** Pre- and post-values of leptin, adiponectin, ghrelin, CCK, GLP-1, GIP, PYY, and body composition at pre-and post-intervention.

	Control		TRT		CRT		IRT		Partial eta squared	*p* values Time*group
	Pre	Post	Pre	Post	Pre	Post	Pre	Post		
Weight (kg)	93.8 ± 2.0	93 ± 2.2	92.9 ± 2.8	90.8 ± 1.8	92.4 ± 1.9	88.8 ± 1.6*	93.7 ± 1.9	87.0 ± 2.1*	0.42	0.000
BMI (kg/m2)	32.9 ± 1.4	32.6 ± 1.4	32.4 ± 1.4	31.7 ± 0.8	33 ± 1.2	31.7 ± 1.2	33.1 ± 0.7	30.7 ± 0.9*	0.42	0.000
Fat (kg)	30.5 ± 1.0	30.5 ± 1.1	29.6 ± 0.7	28.5 ± 0.8	29.9 ± 1.1	27.2 ± 0.8*	30.4 ± 1.0	26.8 ± 1.0*	0.51	0.000
SBP (mmHg)	127 ± 2.35	128 ± 1.75	127 ± 2.0	124 ± 1.91	128 ± 2.2	120 ± 3.0*	127 ± 1.42	118 ± 2.46* #	0.85	0.000
DBP (mmHg)	73.8 ± 6.6	75.0 ± 7.1	71.6 ± 6.4	70.0 ± 5.1	72.8 ± 5.9	68.2 ± 4.7	76.0 ± 6.3	67.6 ± 6.2	0.64	0.000
Leptin (ng/mL)	27.9 ± 3.7	32.7 ± 4.6	27.9 ± 5.7	22.9 ± 3.6*	28.2 ± 3.7	13.5 ± 5.5*#	29.6 ± 8.2	12.2 ± 3.5*#	0.59	0.000
Adiponectin (ng/ml)	5.82 ± 1.13	5.69 ± 1.13	6.48 ± 1.25	8.43 ± 0.98*	6.58 ± 0.57	10.8 ± 1.42*#	6.64 ± 0.62	12.0 ± 1.0*#	0.66	0.000
Ghrelin (pg/ml)	662 ± 20	673 ± 20	659 ± 20	779 ± 17*	650 ± 14	870 ± 11*#	669 ± 31	866 ± 18*#	0.91	0.000

*Data presented as Mean (±SD). BMI, Body mass index; SBP, Systolic blood pressure; DBP, Diastolic blood pressure; CCK, Cholecystokinin; GLP-1, Glucagon-like peptide-1; GIP, Glucose-Dependent Insulinotropic Polypeptide; PYY, Peptide YY; TRT, traditional resistance training; CRT, Circuit resistance training; IRT, Interval resistance training*.

* Indicates significant differences compared to the control group (*p* < 0.05).

# Indicated significant differences between training modes (*p* < 0.05).

### Leptin, Adiponectin, Ghrelin, GLP-1, PYY, and CCK

**Figure 2:**
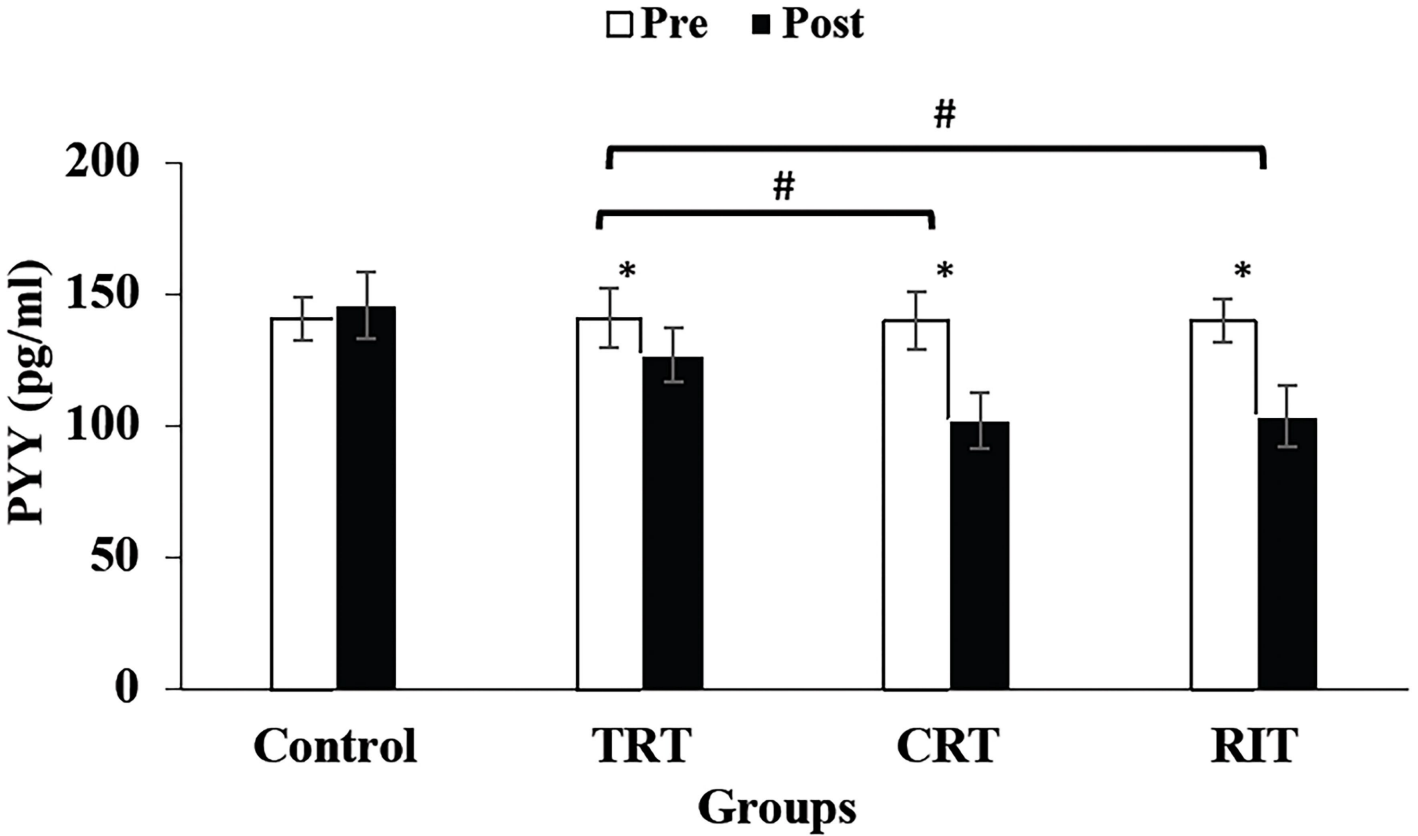
Pre- and post-training values (mean ± SD) for Peptide YY in TRT, CRT, IRT, and groups. ^*^Indicates significant differences from the control group (*p* < 0.05). ^#^Indicated significant differences between training protocols (*p* < 0.05).

**Figure 3:**
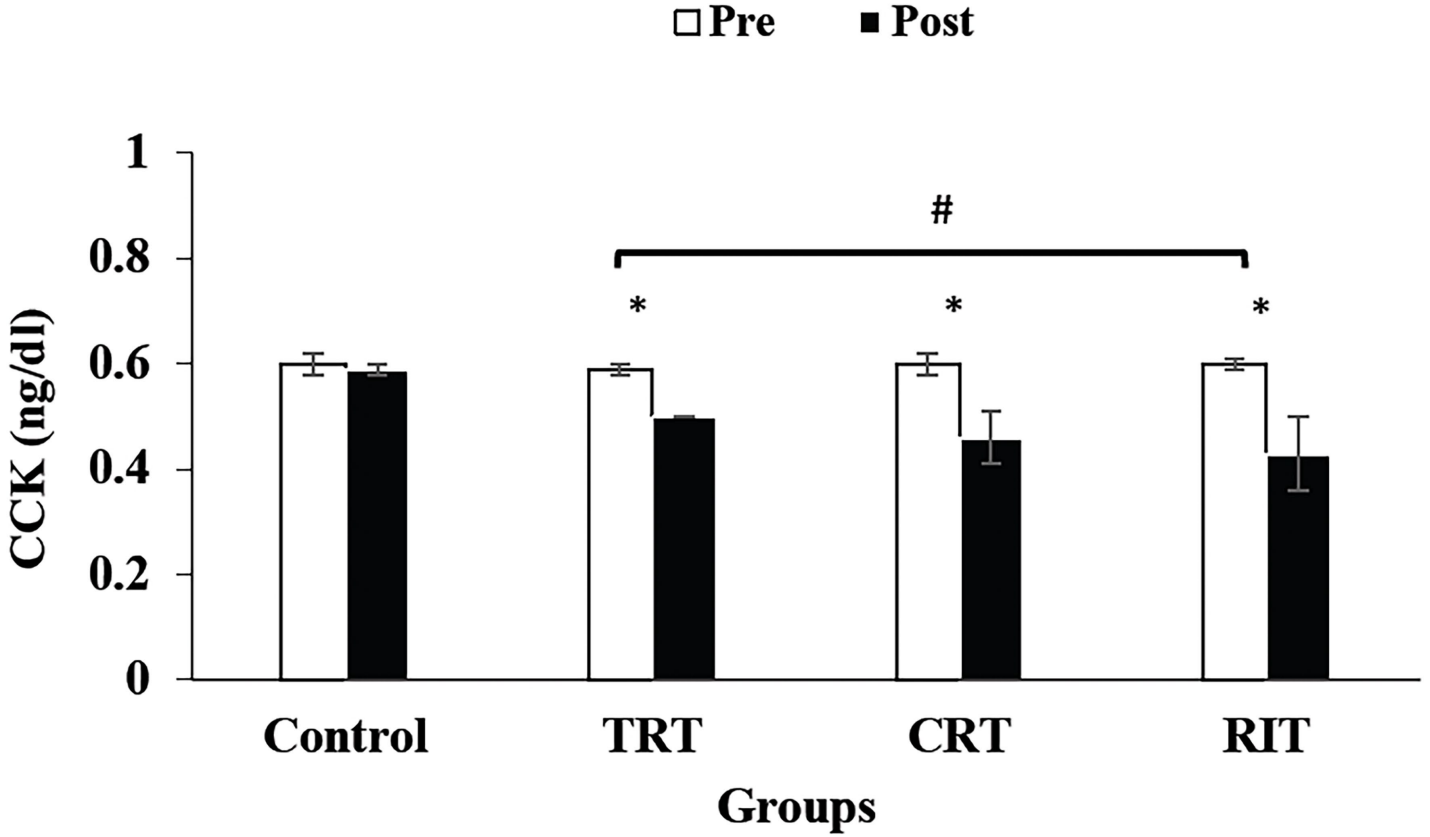
Pre- and post-training values (mean ± SD) for Cholecystokinin in TRT, CRT, and IRT groups. ^*^Indicates significant differences from the control protocols (*p* < 0.05). ^#^Indicated significant differences between training groups (*p* < 0.05).

**Figure 4:**
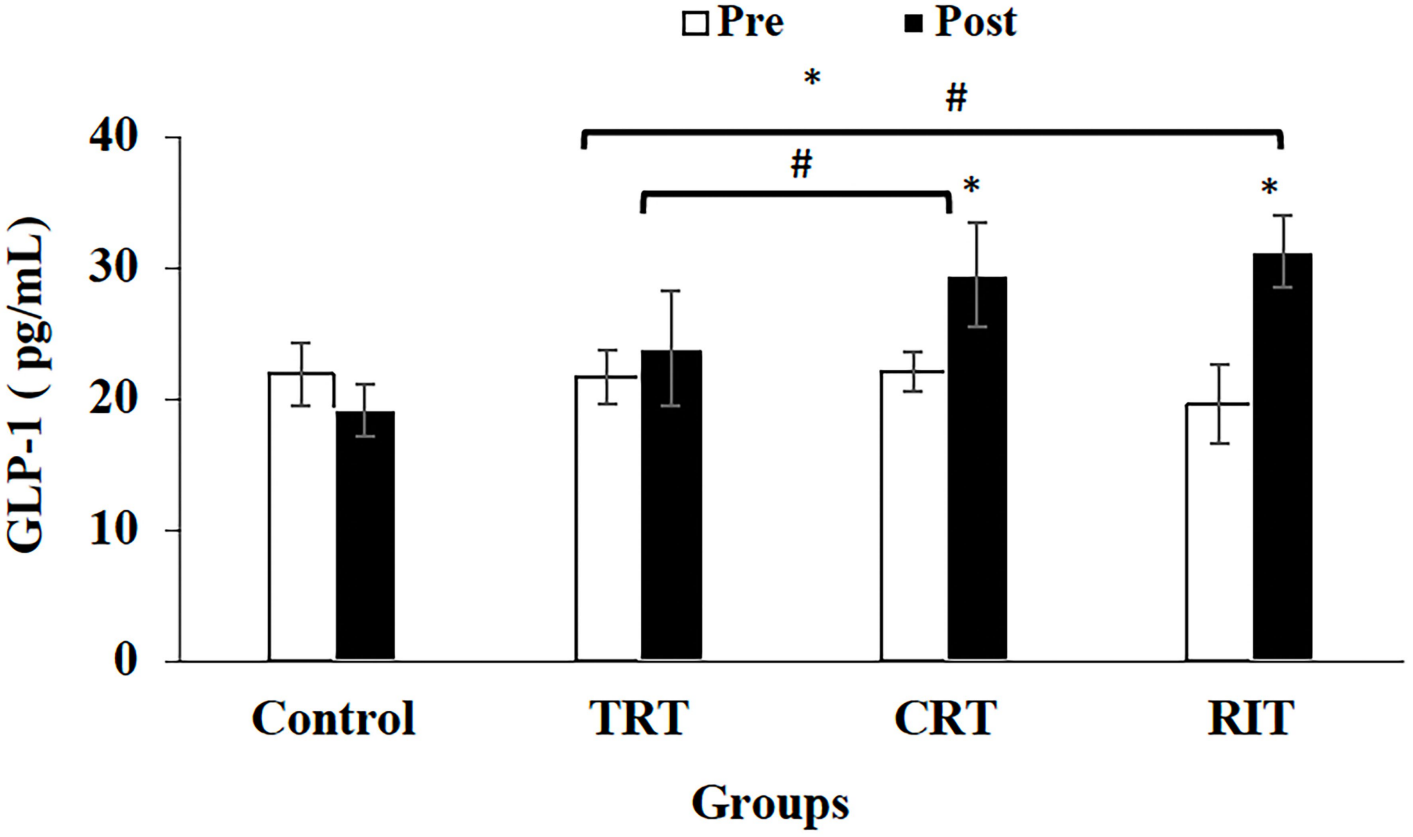
Pre- and post-training values (mean ± SD) for Glucagon-like peptide-1 in TRT, CRT, and IRT groups. ^*^Indicates significant differences from the control group (*p* < 0.05). ^#^Indicated significant differences between training protocols (*p* < 0.05).

**Figure 5:**
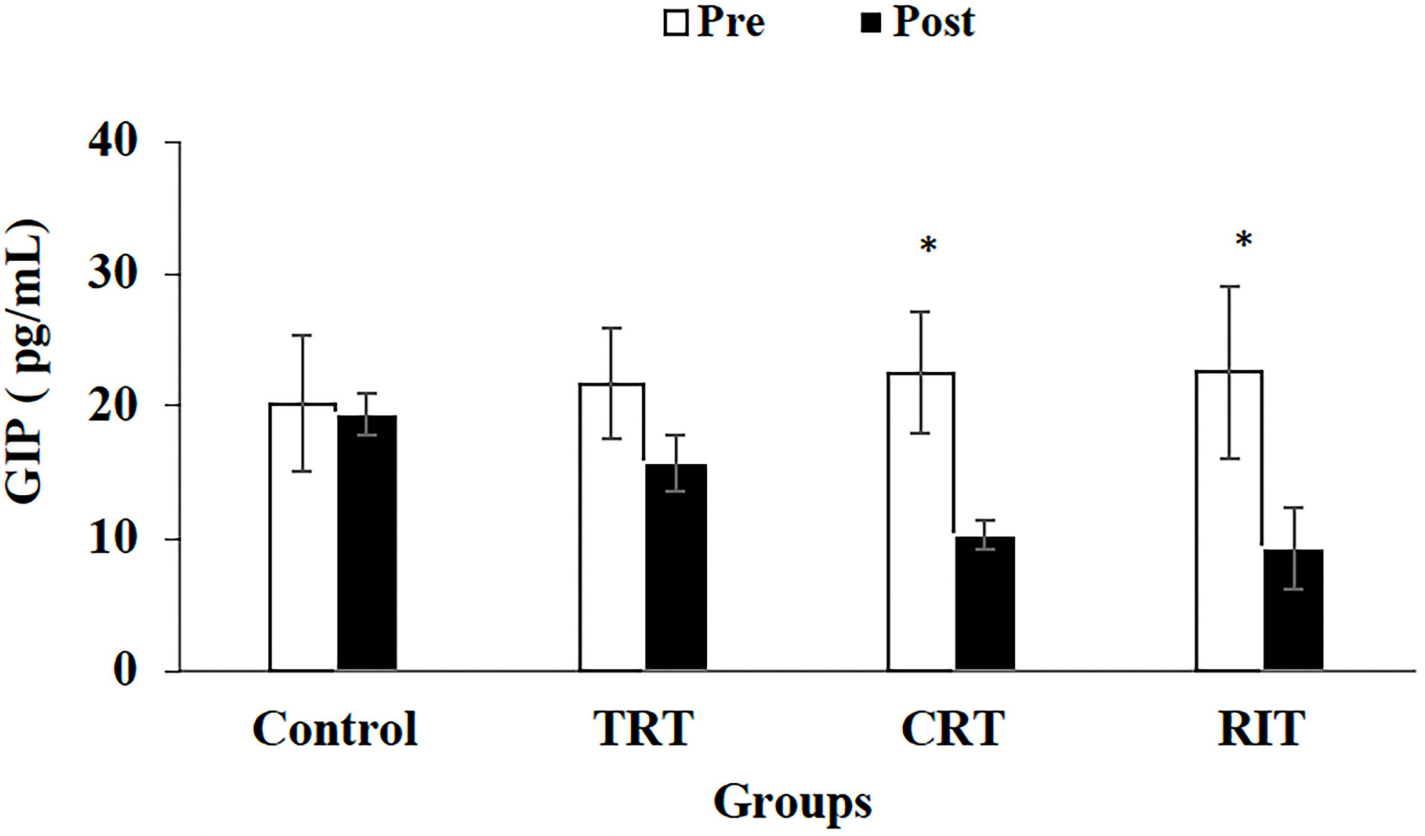
Pre- and post-training values (mean ± SD) for Glucose-Dependent Insulinotropic Polypeptide in TRT, CRT, and IRT groups. ^*^Indicates significant differences from the control group (*p* < 0.05). ^#^Indicated significant differences between training protocols (*p* < 0.05).

The plasma level of leptin and PYY were decreased significantly in all three resistance training groups compared to the control group (*p* < 0.05; Table 3; Figure 2). However, these decreases were greater in CRT and IRT groups compared to the TRT group (*p* < 0.05; Table 3; Figure 2). The plasma level of ghrelin was increased significantly in all three resistance training groups compared to the control group (*p* < 0.05; Table 3). However, these increases were greater in CRT and IRT groups compared to the TRT group (*p* < 0.05; Table 3). CCK significantly reduced in all resistance training groups compared to the control group (*p* < 0.05; Figure 3). However, the decrease was more significant in the IRT group compared to the TRT group (*p* < 0.05; Figure 3). Our results indicated that GLP-1 increased significantly in both CRT and IRT groups than TRT and control groups (*p* < 0.05). Nevertheless, no significant change was observed in the TRT group compared to the control group (*p* < 0.05; Figure 4). Glucose-dependent insulinotropic polypeptide (GIP) decreased significantly only in CRT and IRT groups compared to the control group (*p* < 0.05), but not in the TRT group (*p* < 0.05; Figure 5). Adiponectin levels increased significantly in all resistance training groups compared to the control group (*p* < 0.05). However, these increases were significantly greater in CRT and RIT groups than TRT group (*p* < 0.05; Table 3).

## Discussion

The current study results showed that fat mass, body weight, and body mass index improved significantly after all three different resistance training interventions. Appetite-regulating hormones have been implicated in regulating exercise-induced calorie intake and have been the subject of much research. Our study showed that all three resistance training interventions decreased leptin, CCK, GIP, and increased ghrelin, adiponectin, PYY, and GLP-1 levels compared to the control group. To the best of our knowledge, this is the first supervised randomized control trial on the effect of different modes of resistance training interventions on the appetite-related hormones in males with obesity.

Ghrelin, also known as the hormone of hunger, is a peptide and a stomach-derived orexigenic hormone produced by endocrine cells of the gastric mucosa and recognized as the endogenous ligand of the orphan growth hormone secretagogue receptor. It plays a major role in food intake and appetite regulation. In our study, body weight decreased, and ghrelin increased. Similarly, Leidy et al. (2007) reported a reduction in body weight and an increase in ghrelin concentrations during the day after 3-month aerobic exercise five times per week at 70–80% HRmax and a diet intervention (55% carbohydrates, 30% fat, and 15% protein) in young females with normal weight. However, the authors were unable to determine whether the observed changes came from training, the dietary intervention, or the associated weight loss (Leidy et al., 2007). Some studies have reported increased concentrations of ghrelin after moderate-intensity training (Martins et al., 2007; Kelishadi et al., 2008; Hagobian et al., 2009; Konopko-Zubrzycka et al., 2009; Ueda et al., 2013), while some reported decreased (Kraemer et al., 2004; Ghanbari-Niaki, 2006; Ballard et al., 2009) or no changes (King, 2010; Guelfi et al., 2013; Martins et al., 2017). The intensity of physical activity could play an essential role in ghrelin secretion *via* catecholamine-related mechanisms (Zouhal et al., 2013). However, to date, the response of ghrelin following highly intensive training known to increase catecholamine secretions in both normal-weight individuals and those with obesity has not been investigated. These differences can be explained mainly by the different training interventions, such as training duration, training modalities (e.g., running, walking, or cycling), and the intensity of training used in these studies and others. Also, the increase in ghrelin levels observed in individuals with overweight or obesity may reflect the short-term compensatory effect on appetite stimulation against weight loss (Leidy et al., 2007).

The hormone leptin, which is secreted from adipose tissue and is one of the environmental signals of food intake and energy expenditure, seems to play a role in the long-term regulation of energy homeostasis. The decrease of leptin after weight loss can also reduce its appetite suppressant response and increase appetite (Kissileff et al., 2012). In line with our study that observed the plasma level of leptin were decreased significantly in all three resistance training groups compared to the control group, some studies have reported a decrease in leptin concentration after chronic and acute exercise (Fatouros et al., 2005; Fazelifar et al., 2013; Martins et al., 2013; Ghaderi and Azizbeigi, 2014; Hopkins et al., 2014), while others found no change in leptin levels (Zafeiridis et al., 2003; Lau et al., 2010; Ferdosi and Asad, 2012; Yi et al., 2013; Nuri et al., 2016). One of the possible mechanisms to justify a decrease in serum leptin levels during resistance training can be related to the reduction of body fat and the increase of muscle mass, which increases the energy expenditure at rest and thus reduces leptin secretion in individuals who are overweight or obesity (Ara et al., 2006). Another possible reason could be the increase in ghrelin levels in our study. It has been reported that the low level of ghrelin was associated with high blood pressure and leptin (Pöykkö et al., 2003).

Another finding of our study was an increase in adiponectin levels. Similarly, some of the previous studies have shown increases in adiponectin levels (Fatouros et al., 2005; Brooks et al., 2007; Olson et al., 2007) or decreases (Gondim et al., 2015; Gastebois et al., 2016), or no changes (Ahmadizad et al., 2007; Lau et al., 2010) after exercise training. A recent systematic review (Bouassida et al., 2010) reported that an increase in the level of physical fitness caused by exercise training results in the reduction of fat mass and body mass which are associated with increases in resting adiponectin levels. Similarly, in our study, body fat mass was significantly reduced in all resistance interventions groups. One of the possible mechanisms of increasing adiponectin levels after resistance training is to improve insulin resistance (Ciroma et al., 2017) which is caused by increased oxidation of fatty acids and inhibition of hepatic glucose production (Lihn et al., 2005). Increased adiponectin with exercise interventions, such as resistance training, can increase AMP-activated protein kinase (AMPK) and their associated signal cascade to improve insulin sensitivity and increase the expression of proteins involved in lipid transport and oxidation (Tomas et al., 2004). Tomas et al. (2004) showed that the activity of AMPK is associated with intensity and muscle mass involved in the exercise (Tomas et al., 2004). Therefore, individuals who use higher intensity and more muscle mass during exercise may need more adiponectin to regulate metabolic flow (Racil et al., 2013). In our study, the energy consumption and also serum adiponectin levels increased significantly. This increase is due to the decrease in rest between sets, which may have increased training intensity in the IRT and CRT groups compared to the TRT group.

There are challenges in interpreting adiponectin changes in response to exercise because of differences in the sex of participants in the studies, initial body composition, separating fat loss from exercise-related changes, and different methods of measuring adiponectin.

Glucagon-like peptide-1 is an intestinal hormone secreted in response to food intake. It stimulates insulin secretion by the β cells and reduces glucagon secretion in response to a meal, decreasing hepatic glucose production (Hallworth et al., 2017). The GLP-1 is consecrated with peptide YY, an anorectic hormone. The secretion of peptide YY is proportional to the number of dietary fats ingested during food intake (Hallworth et al., 2017). The GIP is secreted from K cells in the small intestine, and increased secretion has been reported in obesity (Elahi et al., 1984; Drucker, 2007), impaired glucose tolerance (IGT; Salera et al., 1982), and type 2 diabetes (Elahi et al., 1984). The GLP-1 and GIP are collectively called incretins and regulate nearly 60% of postprandial insulin secretion (Drucker, 2007). Our study showed a significant increase in GLP-1 and a significant decrease in PYY and GIP in all three resistance intervention groups compared to the control group. However, these changes were more in the two groups of IRT and CRT.

It has been well established that exercise reduces circulating insulin levels and improves the insulin response to hyperglycemia (Kirwan et al., 1993). It has been suggested that the reductions in plasma insulin following exercise may be due to modulation of the gastroenteropancreatic hormones (Blom et al., 1985) that function primarily to maintain energy balance. Exercise creates a negative energy balance, and different mechanisms maintain energy homeostasis: short-term regulation *via* gut hormones, including GIP, GLP-1, and peptide tyrosine–tyrosine (PYY), and longer-term regulation *via* insulin (Drucker, 2007). This is well illustrated by Gibbons et al. (2017), who demonstrated recently that individuals with overweight and obesity who lost weight (responders to exercise) after a 12-week supervised aerobic intervention (70% of the individual’s HRmax, five times per week) displayed an elevated postprandial rise in GLP-1 and total PYY, and a greater suppression in acylated ghrelin compared to non-responders (Gibbons et al., 2017). Adding support to the notion that changes in body fat could be the key to the changes in appetite hormones as a study showed that greater weight loss in response to high-intensity interval training was associated with greater post-training increases in fasting acylated ghrelin (Martins et al., 2017). In contrast, Martins et al. (2017) (Martins et al., 2017) did not find these relationships with concentrations of PYY or GLP-1. Other studies have shown unchanged fasting and postprandial concentrations of acylated ghrelin (Guelfi et al., 2013), PYY (Blom et al., 1985; Rosenkilde et al., 2013; Larsen et al., 2017), and GLP-1 (Martins et al., 2010; Larsen et al., 2017), despite reductions in body weight after aerobic training. Guelfi et al. (2013) also showed that a reduction in fat mass after resistance training (3 sets of 10 repetitions at 75% 1RM progressed to 4 sets of 8 repetitions at 85% 1RM by the end of the intervention) did not coincide with changes in acylated ghrelin, PYY, or Pancreatic polypeptide concentrations in men with overweight/obesity (Guelfi et al., 2013). Additionally, another study in individuals with obesity reported training-induced elevations in fasting and postprandial Pancreatic polypeptide concentrations in the absence of body fat loss (Kanaley et al., 2014). Reasons for these discrepancies are unclear, and further studies are needed to clarify the variations in appetite-related hormones after exercise training that participants’ body fat could modulate.

When highly acidic food enters the small intestine, cholecystokinin is an anorexigenic hormone secreted by the duodenal and jejunal mucosa. Studies have shown that reduced levels of cholecystokinin may contribute to a reduced feeling of fullness and make it more difficult for some individuals with obesity to lose weight (Little et al., 2005). Cholecystokinin production is impaired (reduced) in individuals with obesity who are experiencing bodyweight reduction (Sumithran et al., 2011). Studies of the effect of exercise and physical activity on cholecystokinin levels are very limited and controversial. For example, plasma cholecystokinin levels increased significantly following intensive training (cycling three times for 4 weeks at 70 to 85% of the HRmax) or hypoxia (Bailey et al., 2000, 2001). Conversely, cholecystokinin levels were decreased in healthy untrained volunteers runners after aerobic exercise (30 min at 70% of vo_2max_; Ströhle et al., 2006) but were unchanged in another exercise study (Ohta et al., 1994). Interestingly, Martins et al. indicated that a 12-week supervised aerobic training (5times per week, 75% HRmax) in individuals with obesity-induced a mean decrease in body weight of 3.5 kg (from 96.2 to 92.7 kg) but had no significant effect on fasting or postprandial cholecystokinin concentrations (Martins et al., 2013). To date, studies on the impact of acute exercise on cholecystokinin levels seem to have been conducted only in normal-weight individuals. These studies reported an increase in cholecystokinin levels immediately after training and for up to 2 h after exercise (Bailey et al., 2001; Sliwowski et al., 2001). These significant increases in cholecystokinin levels are associated with suppressed feelings of hunger during the hours following exercise (Schubert et al., 2014). However, the limited data in this area cannot be generalized to include the effect of exercise and physical activity on cholecystokinin levels in individuals with obesity.

Our results showed that the increase in ghrelin, GLP-1 and decrease in leptin, CCK, GIP, and PYY could be a body’s compensatory mechanism in response to increased energy consumption for the desire to eat more. In other words, an increase in appetite-regulating hormones is likely to act as a compensatory mechanism to return the body weight to the regulated point. The weight loss observed in the groups, in addition to appetite hormones, can also be attributed to changes in adipokines, such as leptin and adiponectin. The finding confirmed previous studies that showed a decrease in leptin and the increase in adiponectin was consistent with the reduction in fat mass (Fatouros et al., 2005; Kissileff et al., 2012; Fazelifar et al., 2013).

We acknowledge that there were some limitations in this study. Firstly, we did not measure the calorie expenditure during the training, and calorie intake during the interventions, which are vital in weight loss; these data could help to interpret the findings with accuracy. Secondly, the gut peptides (GIP, CCK, PYY, and GLP-1) increased in the postprandial phase while we measured their fasting levels, which indicates the chronic effects of an intervention on these peptides. In addition, levels of these peptides increase in the postprandial phase depending on the quality and quantity of food. Finally, we did not measure other adipokines and myokines affecting weight loss. Therefore, we suggest that future research will investigate the gut peptides levels in the postprandial phase before and after a period of resistance training. In addition, future research is needed to measure the levels of other adipokines (e.g., resistin) and myokines (e.g., irisin) that affect weight loss.

## Conclusion

According to the results of this study, the increase in appetite due to adaptation to different modules of resistance training is due to the rise in related hormones. Although the change in hormones was greater in the two groups of resistance training (IRT, CRT), but increase in appetite was observed in all three resistance intervention groups. Therefore, according to the above findings, it is suggested that individuals who complain of anorexia, considering that the hormones tested increase appetite, can use resistance training, especially IRT and CRT training to increase appetite.

## Data Availability Statement

The raw data supporting the conclusions of this article will be made available by the authors, without undue reservation.

## Ethics Statement

The studies involving human participants were reviewed and approved by Research and Ethics Committee of the Islamic Azad University (Ethics code: IR-IAU1397-16). The patients/participants provided their written informed consent to participate in this study.

## Author Contributions

AA, MH, HA, MS, AG-N, AN-H, SA-S, AM, MK, KJ, TV, AS, and HZ whose names appear on the submission made substantial contributions to the conception or design of the work, or the acquisition, analysis, or interpretation of data, drafted the work or revised it critically for important intellectual content, and approved the version to be published. AA, MH, HA, MS, AG-N, AN-H, SA-S, AM, MK, KJ, TV, AS, and HZ agree to be accountable for all aspects of the work in ensuring that questions related to the accuracy or integrity of any part of the work are appropriately investigated and resolved. All authors contributed to the article and approved the submitted version.

## Funding

The authors acknowledge the support (publications fees) of the Kennesaw State University, United States.

## Conflict of Interest

The authors declare that the research was conducted in the absence of any commercial or financial relationships that could be construed as a potential conflict of interest.

## Publisher’s Note

All claims expressed in this article are solely those of the authors and do not necessarily represent those of their affiliated organizations, or those of the publisher, the editors and the reviewers. Any product that may be evaluated in this article, or claim that may be made by its manufacturer, is not guaranteed or endorsed by the publisher.

## References

[ref1] Ahmadizad S. Haghighi A. H. Hamedinia M. R. (2007). Effects of resistance versus endurance training on serum adiponectin and insulin resistance index. Eur. J. Endocrinol. 157, 625–631. doi: 10.1530/EJE-07-0223, PMID: 17984242

[ref2] Ara I. Perez-Gomez J. Vicente-Rodríguez G. Chavarren J. Dorado C. Calbet J. (2006). Serum free testosterone, leptin and soluble leptin receptor changes in a 6-week strength-training programme. Br. J. Nutr. 96, 1053–1059. doi: 10.1017/BJN20061956 17181880

[ref3] Baechle T. R Earle R. W. (2008). Essentials of Strength Training and Conditioning. United Kingdom: Human kinetics.

[ref4] Bailey D. M. Davies B. Castell L. M. Newsholme E. A. Calam J. (2001). Physical exercise and normobaric hypoxia: independent modulators of peripheral cholecystokinin metabolism in man. J. Appl. Physiol. 90, 105–113. doi: 10.1152/jappl.2001.90.1.105, PMID: 11133899

[ref5] Bailey D. M. Davies B. Milledge J. S. Richards M. Williams S. Jordinson M.. (2000). Elevated plasma cholecystokinin at high altitude: metabolic implications for the anorexia of acute mountain sickness. High Alt. Med. Biol. 1, 9–23. doi: 10.1089/152702900320649, PMID: 11258590

[ref6] Balaguera-Cortes L. Wallman K. E. Fairchild T. J. Guelfi K. J. (2011). Energy intake and appetite-related hormones following acute aerobic and resistance exercise. Appl. Physiol. Nutr. Metab. 36, 958–966. doi: 10.1139/h11-121, PMID: 22111518

[ref7] Ballard T. P. Melby C. L. Camus H. Cianciulli M. Pitts J. Schmidt S.. (2009). Effect of resistance exercise, with or without carbohydrate supplementation, on plasma ghrelin concentrations and postexercise hunger and food intake. Metabolism 58, 1191–1199. doi: 10.1016/j.metabol.2009.03.018 19497597

[ref8] Berthoud H.-R. Morrison C. (2008). The brain, appetite, and obesity. Annu. Rev. Psychol. 59, 55–92. doi: 10.1146/annurev.psych.59.103006.093551 18154499

[ref9] Blom P. Høstmark A. Flaten O. Hermansen L. (1985). Modification by exercise of the plasma gastric inhibitory polypeptide response to glucose ingestion in young men. Acta Physiol. Scand. 123, 367–368. doi: 10.1111/j.1748-1716.1985.tb07602.x, PMID: 3904333

[ref10] Bouassida A. Chamari K. Zaouali M. Feki Y. Zbidi A. Tabka Z. (2010). Review on leptin and adiponectin responses and adaptations to acute and chronic exercise. Br. J. Sports Med. 44, 620–630. doi: 10.1136/bjsm.2008.046151, PMID: 18927166

[ref11] Brooks N. Layne J. E. Gordon P. L. Roubenoff R. Nelson M. E. Castaneda-Sceppa C. (2007). Strength training improves muscle quality and insulin sensitivity in Hispanic older adults with type 2 diabetes. Int. J. Med. Sci. 4, 19–27. doi: 10.7150/ijms.4.19 PMC1752232 17211497

[ref12] Brzycki M. (1993). Strength testing—predicting a one-rep max from reps-to-fatigue. J. Physical Educ. Recreat. Dance 64, 88–90. doi: 10.1080/07303084.1993.10606684

[ref13] Ciroma F. Ayo J. Mohammed A. Akor-Dewu M. Kana M. Kase S. (2017). Association between adiponectin, serum lipids and obesity in a university setting in Nigeria. Niger. J. Physiol. Sci. 32, 69–74. PMID: 29134980

[ref14] Douglas J. A. King J. A. Clayton D. J. Jackson A. Sargeant J. A. Thackray A. E.. (2017). Acute effects of exercise on appetite, ad libitum energy intake and appetite-regulatory hormones in lean and overweight/obese men and women. Int. J. Obes. 41, 1737–1744. doi: 10.1038/ijo.2017.181, PMID: 28769121 PMC5729348

[ref15] Drucker D. J. (2007). The role of gut hormones in glucose homeostasis. J. Clin. Invest. 117, 24–32. doi: 10.1172/JCI30076, PMID: 17200703 PMC1716213

[ref16] Elahi D. Andersen D. K. Muller D. C. Tobin J. D. Brown J. C. Andres R. (1984). The enteric enhancement of glucose-stimulated insulin release: the role of GIP in aging, obesity, and non-insulin-dependent diabetes mellitus. Diabetes 33, 950–957. doi: 10.2337/diab.33.10.950, PMID: 6383904

[ref17] Fatouros I. Tournis S. Leontsini D. Jamurtas A. Sxina M. Thomakos P.. (2005). Leptin and adiponectin responses in overweight inactive elderly following resistance training and detraining are intensity related. J. Clin. Endocrinol. Metab. 90, 5970–5977. doi: 10.1210/jc.2005-0261, PMID: 16091494

[ref18] Fazelifar S. Ebrahim K. Sarkisian V. (2013). Effect of exercise training and detraining on serum leptin levels in obese young boys. Med. Sport. 66, 325–337.

[ref19] Ferdosi M. H. Asad M. R. (2012). The effect of endurance, resistance and concurrent trainings on plasma leptin levels of non-athlete males. Procedia Soc. Behav. Sci. 46, 311–315. doi: 10.1016/j.sbspro.2012.05.112

[ref20] Franz M. J. VanWormer J. J. Crain A. L. Boucher J. L. Histon T. Caplan W.. (2007). Weight-loss outcomes: a systematic review and meta-analysis of weight-loss clinical trials with a minimum 1-year follow-up. J. Am. Diet. Assoc. 107, 1755–1767. doi: 10.1016/j.jada.2007.07.017, PMID: 17904936

[ref21] Gastebois C. Villars C. Drai J. Canet-Soulas E. Blanc S. Bergouignan A.. (2016). Effects of training and detraining on adiponectin plasma concentration and muscle sensitivity in lean and overweight men. Eur. J. Appl. Physiol. 116, 2135–2144. doi: 10.1007/s00421-016-3466-z, PMID: 27632382

[ref22] Ghaderi M. Azizbeigi K. (2014). Hormonal responses to acute resistance exercise after branched-chain amino acids supplementation. Int. Med. J. 22, 1–5.

[ref23] Ghanbari-Niaki A. (2006). Ghrelin and glucoregulatory hormone responses to a single circuit resistance exercise in male college students. Clin. Biochem. 39, 966–970. doi: 10.1016/j.clinbiochem.2006.05.009, PMID: 16979150

[ref24] Gibbons C. Blundell J. E. Caudwell P. Webb D.-L. Hellström P. M. Näslund E.. (2017). The role of episodic postprandial peptides in exercise-induced compensatory eating. J. Clin. Endocrinol. Metab. 102, 4051–4059. doi: 10.1210/jc.2017-00817, PMID: 28938473 PMC5673273

[ref25] Gondim O. S. Camargo V. T. N. Gutierrez F. A. Martins P. F. O. Passos M. E. P. Momesso C. M.. (2015). Benefits of regular exercise on inflammatory and cardiovascular risk markers in normal weight, overweight and obese adults. PLoS One 10:e0140596. doi: 10.1371/journal.pone.0140596, PMID: 26474157 PMC4608693

[ref26] Guelfi K. J. Donges C. E. Duffield R. (2013). Beneficial effects of 12 weeks of aerobic compared with resistance exercise training on perceived appetite in previously sedentary overweight and obese men. Metabolism 62, 235–243. doi: 10.1016/j.metabol.2012.08.002, PMID: 22959499

[ref27] Hagobian T. A. Sharoff C. G. Stephens B. R. Wade G. N. Silva J. E. Chipkin S. R.. (2009). Effects of exercise on energy-regulating hormones and appetite in men and women. Am. J. Phys. Regul. Integr. Comp. Phys. 296, R233–R242. doi: 10.1152/ajpregu.90671.2008 PMC2643988 19073905

[ref28] Hallworth J. R. Copeland J. L. Doan J. Hazell T. J. (2017). The effect of exercise intensity on total PYY and GLP-1 in healthy females: a pilot study. J. Nutr. Metab. 2017, 1–7. doi: 10.1155/2017/4823102, PMID: 28286674 PMC5327759

[ref29] Haththotuwa R. N. Wijeyaratne C. N. Senarath U. (2020). “Worldwide epidemic of obesity,” in Obesity and Obstetrics. *2nd Edn*. eds. Mahmood T. A. Arulkumaran S. Chervenak F. A. (Netherlands: Elsevier), 3–8.

[ref30] Hopkins M. Blundell J. E. (2016). Energy balance, body composition, sedentariness and appetite regulation: pathways to obesity. Clin. Sci. 130, 1615–1628. doi: 10.1042/CS20160006, PMID: 27503946

[ref31] Hopkins M. Gibbons C. Caudwell P. Webb D.-L. Hellström P. M. Näslund E.. (2014). Fasting leptin is a metabolic determinant of food reward in overweight and obese individuals during chronic aerobic exercise training. Int. J. Endocrinol. 2014, 1–8. doi: 10.1155/2014/323728, PMID: 24734042 PMC3966321

[ref32] Kanaley J. A. Heden T. D. Liu Y. Whaley-Connell A. T. Chockalingam A. Dellsperger K. C.. (2014). Short-term aerobic exercise training increases postprandial pancreatic polypeptide but not peptide YY concentrations in obese individuals. Int. J. Obes. 38, 266–271. doi: 10.1038/ijo.2013.84, PMID: 23736355 PMC3773306

[ref33] Kelishadi R. Hashemipour M. Mohammadifard N. Alikhassy H. Adeli K. (2008). Short-and long-term relationships of serum ghrelin with changes in body composition and the metabolic syndrome in prepubescent obese children following two different weight loss programmes. Clin. Endocrinol. 69, 721–729. doi: 10.1111/j.1365-2265.2008.03220.x, PMID: 18284632

[ref34] Kim D. Hou W. Wang F. Arcan C. (2019). Peer reviewed: factors affecting obesity and waist circumference Among US adults. Prev. Chronic Dis. 16:E02. doi: 10.5888/pcd16.180220, PMID: 30605422 PMC6341820

[ref35] King J. A. (2010). Effects of exercise on appetite, food intake and the gastrointestinal hormones ghrelin and peptide YY. Doctoral dissertation © JA King.

[ref36] Kirwan J. P. Kohrt W. M. Wojta D. M. Bourey R. E. Holloszy J. O. (1993). Endurance exercise training reduces glucose-stimulated insulin levels in 60-to 70-year-old men and women. J. Gerontol. 48, M84–M90. doi: 10.1093/geronj/48.3.M84, PMID: 8482816

[ref37] Kissileff H. R. Thornton J. C. Torres M. I. Pavlovich K. Mayer L. S. Kalari V.. (2012). Leptin reverses declines in satiation in weight-reduced obese humans. Am. J. Clin. Nutr. 95, 309–317. doi: 10.3945/ajcn.111.012385, PMID: 22237063 PMC3260066

[ref38] Konopko-Zubrzycka M. Baniukiewicz A. Wroblewski E. Kowalska I. Zarzycki W. Górska M.. (2009). The effect of intragastric balloon on plasma ghrelin, leptin, and adiponectin levels in patients with morbid obesity. J. Clin. Endocrinol. Metab. 94, 1644–1649. doi: 10.1210/jc.2008-1083, PMID: 19258408

[ref39] Kraemer R. Durand R. Acevedo E. Johnson L. Kraemer G. Hebert E.. (2004). Rigorous running increases growth hormone and insulin-like growth factor-I without altering ghrelin. Exp. Biol. Med. 229, 240–246. doi: 10.1177/153537020422900304, PMID: 14988516

[ref40] Larsen P. S. Donges C. E. Guelfi K. J. Smith G. C. Adams D. R. Duffield R. (2017). Effects of aerobic, strength or combined exercise on perceived appetite and appetite-related hormones in inactive middle-aged men. Int. J. Sport Nutr. Exerc. Metab. 27, 389–398. doi: 10.1123/ijsnem.2017-0144 28657803

[ref41] Lau P. W. Kong Z. Choi C.-r. Clare C. Chan D. F. Sung R. Y.. (2010). Effects of short-term resistance training on serum leptin levels in obese adolescents. J. Exerc. Sci. Fit. 8, 54–60. doi: 10.1016/S1728-869X(10)60008-1

[ref42] Leidy H. J. Dougherty K. A. Frye B. R. Duke K. M. Williams N. I. (2007). Twenty-four-hour ghrelin is elevated after calorie restriction and exercise training in non-obese women. Obesity 15, 446–455. doi: 10.1038/oby.2007.542, PMID: 17299118

[ref43] Lihn A. Pedersen S. B. Richelsen B. (2005). Adiponectin: action, regulation and association to insulin sensitivity. Obes. Rev. 6, 13–21. doi: 10.1111/j.1467-789X.2005.00159.x, PMID: 15655035

[ref44] Little T. Horowitz M. Feinle-Bisset C. (2005). Role of cholecystokinin in appetite control and body weight regulation. Obes. Rev. 6, 297–306. doi: 10.1111/j.1467-789X.2005.00212.x 16246215

[ref45] Martins C. Aschehoug I. Ludviksen M. Holst J. Finlayson G. Wisloff U.. (2017). High-intensity interval training, appetite, and reward value of food in the obese. Med. Sci. Sports Exerc. 49, 1851–1858. doi: 10.1249/MSS.0000000000001296, PMID: 28398946

[ref46] Martins C. Kulseng B. King N. Holst J. Blundell J. (2010). The effects of exercise-induced weight loss on appetite-related peptides and motivation to eat. J. Clin. Endocrinol. Metab. 95, 1609–1616. doi: 10.1210/jc.2009-2082, PMID: 20150577

[ref47] Martins C. Kulseng B. Rehfeld J. King N. Blundell J. (2013). Effect of chronic exercise on appetite control in overweight and obese individuals. Med. Sci. Sports Exerc. 45, 805–812. doi: 10.1249/MSS.0b013e31827d1618, PMID: 23247700

[ref48] Martins C. Morgan L. M. Bloom S. R. Robertson M. D. (2007). Effects of exercise on gut peptides, energy intake and appetite. J. Endocrinol. 193, 251–258. doi: 10.1677/JOE-06-0030 17470516

[ref49] Moro T. Marcolin G. Bianco A. Bolzetta F. Berton L. Sergi G.. (2020). Effects of 6 weeks of traditional resistance training or high intensity interval resistance training on body composition, aerobic power and strength in healthy young subjects: a randomized parallel trial. Int. J. Environ. Res. Public Health 17:4093. doi: 10.3390/ijerph17114093, PMID: 32521745 PMC7312403

[ref50] Nathanson V. (2013). Revising the declaration of Helsinki. British Med. J. Publish. Group 346:f2837. doi: 10.1136/bmj.f2837, PMID: 23657182

[ref51] Neary N. M. Goldstone A. P. Bloom S. R. (2004). Appetite regulation: from the gut to the hypothalamus. Clin. Endocrinol. 60, 153–160. doi: 10.1046/j.1365-2265.2003.01839.x, PMID: 14725674

[ref52] Nuri R. Moghaddasi M. Darvishi H. Izadpanah A. (2016). Effect of aerobic exercise on leptin and ghrelin in patients with colorectal cancer. J. Cancer Res. Ther. 12, 169–174. doi: 10.4103/0973-1482.155982, PMID: 27072232

[ref53] Ohta M. Ichikawa M. Sazaki N. Okubo K. Miyasaka K. Fujita Y.. (1994). Effect of long-term exercise under restricted-feeding on intestinal content of cholecystokinin and on the pancreas in aging rats. Arch. Gerontol. Geriatr. 18, 43–51. doi: 10.1016/0167-4943(94)90046-9, PMID: 15374312

[ref54] Olson T. P. Dengel D. Leon A. Schmitz K. (2007). Changes in inflammatory biomarkers following one-year of moderate resistance training in overweight women. Int. J. Obes. 31, 996–1003. doi: 10.1038/sj.ijo.0803534, PMID: 17299382

[ref55] Pearson M. J. King N. Smart N. A. (2018). Effect of exercise therapy on established and emerging circulating biomarkers in patients with heart failure: a systematic review and meta-analysis. Arch. Dis. childhood 5:e000819. doi: 10.1136/openhrt-2018-000819, PMID: 30018779 PMC6045761

[ref56] Pöykkö S. M. Kellokoski E. Hörkkö S. Kauma H. Kesäniemi Y. A. Ukkola O. (2003). Low plasma ghrelin is associated with insulin resistance, hypertension, and the prevalence of type 2 diabetes. Diabetes 52, 2546–2553. doi: 10.2337/diabetes.52.10.2546, PMID: 14514639

[ref57] Racil G. Ounis O. B. Hammouda O. Kallel A. Zouhal H. Chamari K.. (2013). Effects of high vs. moderate exercise intensity during interval training on lipids and adiponectin levels in obese young females. Eur. J. Appl. Physiol. 113, 2531–2540. doi: 10.1007/s00421-013-2689-5, PMID: 23824463

[ref58] Redman L. M. Heilbronn L. K. Martin C. K. Alfonso A. Smith S. R. Ravussin E. (2007). Effect of calorie restriction with or without exercise on body composition and fat distribution. J. Clin. Endocrinol. Metab. 92, 865–872. doi: 10.1210/jc.2006-2184, PMID: 17200169 PMC2692618

[ref59] Rocha J. Paxman J. Dalton C. Winter E. Broom D. R. (2016). Effects of a 12-week aerobic exercise intervention on eating behaviour, food cravings, and 7-day energy intake and energy expenditure in inactive men. Appl. Physiol. Nutr. Metab. 41, 1129–1136. doi: 10.1139/apnm-2016-0189, PMID: 27769147

[ref60] Rosenkilde M. Reichkendler M. H. Auerbach P. Toräng S. Gram A. S. Ploug T.. (2013). Appetite regulation in overweight, sedentary men after different amounts of endurance exercise: a randomized controlled trial. J. Appl. Physiol. 115, 1599–1609. doi: 10.1152/japplphysiol.00680.2013, PMID: 24052035

[ref61] Ross R. Dagnone D. Jones P. J. Smith H. Paddags A. Hudson R.. (2000). Reduction in obesity and related comorbid conditions after diet-induced weight loss or exercise-induced weight loss in men. A randomized, controlled trial. Ann. Intern. Med. 133, 92–103. doi: 10.7326/0003-4819-133-2-200007180-00008 10896648

[ref62] Saeidi A. Seifi-Ski-Shahr F. Soltani M. Daraei A. Shirvani H. Laher I.. (2020). Resistance training, gremlin 1 and macrophage migration inhibitory factor in obese men: a randomised trial. Arch. Physiol. Biochem. 1–9. doi: 10.1080/13813455.2020.1856142 [Epub ahead of print] 33370549

[ref63] Salera M. Giacomoni P. Pironi L. Cornia G. Capelli M. Marini A.. (1982). Gastric inhibitory polypeptide release after oral glucose: relationship to glucose intolerance, diabetes mellitus, and obesity. J. Clin. Endocrinol. Metab. 55, 329–336. doi: 10.1210/jcem-55-2-329, PMID: 7045154

[ref64] Schubert M. M. Sabapathy S. Leveritt M. Desbrow B. (2014). Acute exercise and hormones related to appetite regulation: a meta-analysis. Sports Med. 44, 387–403. doi: 10.1007/s40279-013-0120-3, PMID: 24174308

[ref65] Sliwowski Z. Lorens K. Konturek S. Bielanski W. Zoladz J. (2001). Leptin, gastrointestinal and stress hormones in response to exercise in fasted or fed subjects and before or after blood donation. J. Physiol. Pharmacol. 52, 53–70. PMID: 11321513

[ref66] Ströhle A. Feller C. Strasburger C. J. Heinz A. Dimeo F. (2006). Anxiety modulation by the heart? Aerobic exercise and atrial natriuretic peptide. Psychoneuroendocrinology 31, 1127–1130. doi: 10.1016/j.psyneuen.2006.08.003, PMID: 17010527

[ref67] Sumithran P. Prendergast L. A. Delbridge E. Purcell K. Shulkes A. Kriketos A.. (2011). Long-term persistence of hormonal adaptations to weight loss. N. Engl. J. Med. 365, 1597–1604. doi: 10.1056/NEJMoa1105816, PMID: 22029981

[ref68] Tavassoli H. Tofighi A. Hossein Panah F. Hedaytai M. (2014). Appetite and exercise influence of 12 weeks of circuit resistance training on the Nesfatin-1 to Acylated ghrelin ratio of plasma in overweight adolescents. Iran. J. Endocrinol. Metab. 15, 519–526.

[ref69] Thomas D. T. Erdman K. A. Burke L. M. (2016). Position of the academy of nutrition and dietetics, dietitians of Canada, and the American College of Sports Medicine: nutrition and athletic performance. J. Acad. Nutr. Diet. 116, 501–528. doi: 10.1016/j.jand.2015.12.006, PMID: 26920240

[ref70] Thomas S. Reading J. Shephard R. J. (1992). Revision of the physical activity readiness questionnaire (PAR-Q). Can. J. Sport Sci. 17, 338–345. PMID: 1330274

[ref71] Tomas E. Kelly M. Xiang X. Tsao T.-S. Keller C. Keller P.. (2004). Metabolic and hormonal interactions between muscle and adipose tissue. Proc. Nutr. Soc. 63, 381–385. doi: 10.1079/PNS2004356, PMID: 15294059

[ref72] Ueda H. Yagi T. Amitani H. Asakawa A. Ikeda S. Miyawaki S.. (2013). The roles of salivary secretion, brain–gut peptides, and oral hygiene in obesity. Obes. Res. Clin. Pract. 7, e321–e329. doi: 10.1016/j.orcp.2013.05.001, PMID: 24455760

[ref73] Van Gaal L. F. Mertens I. L. Christophe E. (2006). Mechanisms linking obesity with cardiovascular disease. Nature 444, 875–880. doi: 10.1038/nature05487 17167476

[ref74] Yi X. Cao S. Chang B. Zhao D. Gao H. Wan Y.. (2013). Effects of acute exercise and chronic exercise on the liver leptin-AMPK-ACC signaling pathway in rats with type 2 diabetes. J. Diabetes Res. 2013, 1–9. doi: 10.1155/2013/946432, PMID: 24455748 PMC3877642

[ref75] Zafeiridis A. Smilios I. Considine R. V. Tokmakidis S. P. (2003). Serum leptin responses after acute resistance exercise protocols. J. Appl. Physiol. 94, 591–597. doi: 10.1152/japplphysiol.00330.2002, PMID: 12391130

[ref76] Zanchi D. Depoorter A. Egloff L. Haller S. Mählmann L. Lang U. E.. (2017). The impact of gut hormones on the neural circuit of appetite and satiety: a systematic review. Neurosci. Biobehav. Rev. 80, 457–475. doi: 10.1016/j.neubiorev.2017.06.013, PMID: 28669754

[ref77] Zouhal H. Lemoine-Morel S. Mathieu M.-E. Casazza G. A. Jabbour G. (2013). Catecholamines and obesity: effects of exercise and training. Hackney A. Constantini N. (eds) Sports Med. 43, 591–600. doi: 10.1007/s40279-013-0039-8, PMID: 23613311

[ref78] Zouhal H. Saeidi A. Kolahdouzi S. Ahmadizad S. Hackney A. C. Abderrahmane A. B. (2020). “Exercise and training effects on appetite-regulating hormones in individuals with obesity,” in Endocrinology of Physical Activity and Sport. Contemporary Endocrinology (Cham: Humana), 535–562.

[ref79] Zouhal H. Sellami M. Saeidi A. Slimani M. Abbassi-Daloii A. Khodamoradi A.. (2019). Effect of physical exercise and training on gastrointestinal hormones in populations with different weight statuses. Nutr. Rev. 77, 455–477. doi: 10.1093/nutrit/nuz005, PMID: 31125091

